# Observations on the Mode of Curing the Tinea Capitis

**Published:** 1805-12

**Authors:** James Barlow

**Affiliations:** Blackburn, Lancashire


### Observations on the Mode of curing the Tinea Capitis; *by* Mr. James Barlow, *of Blackburn, Lancashire.*

ON reviewing the various remedies which have been adopted for the cure of this disease, both by ancient and modern writers, some of which at times have appeared in your Journal; and having reason to doubt their efficacy, as a sovereign remedy, I am thereby induced to offer to the public, the following mode of treating this obstinate malady. It appears evident, from the numerous and diversified means which have been recommended for this cutaneous affection, that practitioners in medicine must have frequently experienced great difficulty in accomplishing a cure.

The intractableness of most children, when attempted to be controuled or governed by the accustomed mode of treatment, proves, in most instances, a material obstacle in the way of treating this malignant disease; and the quickness with which the hair of the scalp grows in children, has hitherto also, in most instances, rendered every effort ineffectual. It was from a constant failure under these inauspicious circumstances, that led me to adopt the subjoined lotion; and I am happy to say, that by bathing the affected head therewith a few times, morning and evening, and suffering the parts to dry without interruption, the scabs will decorticate and peel off from the scalp, and leave the parts underneath perfectly healed; and this without torturing the patient by either shaving the head, or cutting off the hair. This application I have invariably found to answer (when duly applied) both in children and adults, and in many inveterate cases, even where every other means had been previously used without effect, some of which were of several years standing.

℞. Kali sulphurat (recens preparat) ʒiij. Sapo alb. Hispan. ʒjß. Aq. calcis ℥vijß. Spir. vinos rect ʒij. M. ft. lotio pro tinea capitis.

Might not the above remedy for tinea capitis be efficacious in relieving that dreadful endemic disease, called trichoma, or plica polonica?

*October* 20, 1805.

